# Epicardial Pulsed Field Ablation for Bachmann Bundle–Mediated Atrial Tachycardia: Expanding Indications and Identifying Limitations

**DOI:** 10.1016/j.jaccas.2026.107386

**Published:** 2026-04-15

**Authors:** Johanna B. Tonko

**Affiliations:** National Heart and Lung Institute, Imperial College London, London, United Kingdom

**Keywords:** ablation, atrial tachycardia, electrophysiology

**Figure:**
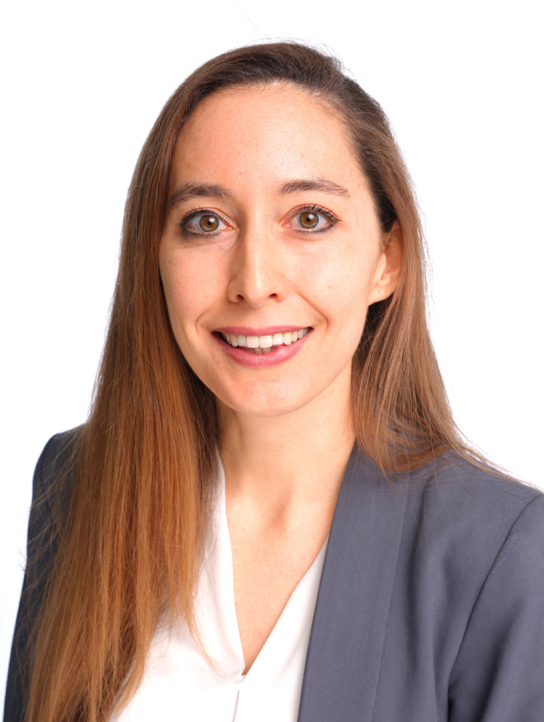

As the principal interatrial conduction pathway ensuring atrial synchrony, Bachmann's bundle, originally described in 1916 by George Bachmann, plays a central role in atrial electrophysiology.^1^^,^^2^ However, beyond its physiological function, it has also been recognized as an important substrate for atrial arrhythmogenesis. Its complex 3-dimensional architecture with epicardial segments may render both diagnosis and catheter-based treatment of focal activity and participation in macro-re-entrant circuits challenging.^3^ Importantly, disruption of Bachmann bundle conduction, whether disease-associated or iatrogenic, has been linked to interatrial delay and the development of atrial tachyarrhythmias,^4^ underscoring the need for ablation strategies that are both effective and anatomically considerate to preserve physiological atrial activation whenever possible.

The limitations of conventional endocardial lesion delivery for successful abolition of the arrhythmia substrate in the presence of epicardial conduction pathways is receiving increasing attention. Although the thin-walled atria have traditionally been considered amenable to transmural lesion formation using radiofrequency (RF) energy, often even with greater concern for collateral damage beyond the myocardial wall, it is now well established that structures such as the vein of Marshall, the septopulmonary bundle, and the Bachmann bundle may form complex 3-dimensional arrhythmia substrates, which can be difficult to eliminate from the endocardium alone because of interposed adipose tissue that electrically insulates the epicardial myocardial bundles.^3^^,^^5^ Ethanol ablation has been proposed to target epicardial connections associated with the vein of Marshall,^6^ but its applicability at other anatomical sites is limited because of a lack of consistent “vascular access.”^7^ Combined endo-epicardial ablation strategies may offer transmural lesion formation^8^ and may gain further traction with the recent development of dedicated tools to facilitate epicardial access,^9^ yet evidence to guide patient selection and choice of energy sources for epicardial atrial ablation procedures remains limited. Alternative approaches to enhance endocardial RF delivery to achieve deeper lesions, such as impedance modulation,^10^ may offer complementary solutions, though again their safety and efficacy in the atria remain to be established.

In view of these limitations, interest has shifted toward non-thermal ablation technologies that may overcome some of the lesion depth and/or anatomical constraints of conventional thermal or chemical ablation approaches. Pulsed field ablation (PFA) has rapidly emerged as a promising ablation modality because of its tissue selectivity and rapid lesion delivery.^11^ Clinical studies have reported its ability to create relatively uniform and transmural lesions while maintaining a favorable safety profile,^12^ raising enthusiasm for its application in anatomically complex substrates beyond pulmonary vein isolation (PVI).

Against this background, the case presented by Jin et al^13^ describes the first reported use of epicardial PFA targeting Bachmann bundle–mediated atrial tachycardia. The procedural findings further highlight both the diagnostic complexity posed by epicardial atrial structures and the ongoing need for improvement and systematic evaluation of PFA delivery in non-PVI settings.

Although the authors have mapped various anatomical structures, the presented mapping data acquired with a circular catheter appear less detailed and dense than what contemporary mapping systems may offer. High-resolution mapping has been shown to be critical for defining complex atrial conduction patterns involving both mitral isthmus^14^^,^^15^ and roof-dependent tachycardias.^7^ If epicardial connections are suspected, a combination of high-density mapping and judicious use of differential pacing maneuvers may be essential to define the exact mechanism and derive a tailored ablation strategy. Current PFA development has largely focused on anatomical ablation for PVI, yet this case highlights the need to fully integrate PFA technology into modern high-resolution mapping workflows for complex atrial tachycardias.

Despite endocardial ablation with PFA, the atrial tachycardia in the present case persisted, underscoring the aforementioned limitations of endocardial energy delivery in the presence of epicardial conduction pathways, including for PFA. Equally, although epicardial PFA application resulted in acute termination of the atrial tachycardia, the subsequent early recurrence serves as a critical reminder of the current limitations of PFA lesion durability. Experimental studies have evaluated lesion depths for various energy delivery protocols, but lesion formation and long-term durability at specific anatomical sites and arrhythmia types in humans remain areas of ongoing research and are still incompletely understood.^16^ The requirement for repeated RF ablation in the presented case indicates that PFA cannot yet be considered a universal solution for interventional arrhythmia treatment.

This observation should not be interpreted as a failure of PFA as a modality, but rather as a reflection of the anatomical complexity of epicardial arrhythmia substrates and the important need for continued refinement of PFA delivery strategies and catheter designs. Optimization and systematic evaluation of site- and arrhythmia-specific PFA parameters, including waveform configuration (monophasic vs biphasic), pulse numbers, width and duration, interpulse delays, as well as electrode designs and catheter-tissue contact assessment, may all be required to improve success rates and lesion durability at both PVI and non-PVI targets.

In conclusion, this case report provides valuable early insights into the use of epicardial atrial PFA while highlighting current limitations and areas of uncertainty. The appeal of epicardial PFA as a tissue selective ablation technology with a favorable safety profile with respect to collateral injury to extracardiac structures is evident, yet the added complexity, uncertain efficacy and durability, and potential risks of epicardial access itself imply that, at present, it may only be justifiable as an investigational bailout strategy in selected cases of highly symptomatic drug-refractory arrhythmias and/or tachycardiomyopathies with clear evidence of epicardial involvement and failure of conventional endocardial strategies.

Future research should focus on prospective comparisons of endocardial vs combined endo-epicardial PFA as well as vs corresponding RF strategies, establish benchmarks for lesion durability for PFA at non-PVI atrial targets, and systematically define optimal site-specific energy delivery parameters. Such studies will be essential to determine the role of PFA in the interventional treatment of complex atrial tachycardias involving epicardial conduction pathways.

## Funding Support and Author Disclosures

Dr Tonko was supported by the Imperial Post-Doctoral, Post-CCT Research Fellowship.
